# Excitonic absorption and defect-related emission in three-dimensional MoS_2_ pyramids†

**DOI:** 10.1039/d1nr06041d

**Published:** 2021-12-17

**Authors:** M. Negri, L. Francaviglia, D. Kaplan, V. Swaminathan, G. Salviati, A. Fontcuberta i Morral, F. Fabbri

**Affiliations:** Institute of Materials, Faculty of Engineering, École Polytechnique Fédérale de Lausanne 1015 Lausanne Switzerland negri.m1@gmail.com; Institute for Materials for Electronics and Magnetism (IMEM-CNR) Parco Area delle Scienze 37/A 43124 Parma Italy; U.S. Army RDECOM-ARDEC, Fuze Precision Armaments and Technology Directorate, Picatinny Arsenal NJ 07806 USA; Department of Physics, Penn State University USA; Institute of Physics, Faculty of Basic Sciences, École Polytechnique Fédérale de Lausanne 1015 Lausanne Switzerland; NEST, Istituto Nanoscienze - CNR, Scuola Normale Superiore Piazza San Silvestro 12 56127 Pisa Italy

## Abstract

MoS_2_ micro-pyramids have demonstrated interesting properties in the fields of photonics and non-linear optics. In this work, we show the excitonic absorption and cathodoluminescence (CL) emission of MoS_2_ micro-pyramids grown by chemical vapor deposition (CVD) on SiO_2_ substrates. The excitonic absorption was obtained at room and cryogenic temperatures by taking advantage of the cathodoluminescence emission of the SiO_2_ substrate. We detected the CL emission related to defect intra-gap states, localized at the pyramid edges and with an enhanced intensity at the pyramid basal vertices. The photoluminescence and absorption analysis provided the Stokes shift of both the A and B excitons in the MoS_2_ pyramids. This analysis provides new insights into the optical functionality of MoS_2_ pyramids. This method can be applied to other 3D structures within the 2D materials family.

## Abstract 2

In this work, we study the excitonic absorption and cathodoluminescence emission of MoS_2_ micro-pyramids grown by chemical vapor deposition on SiO_2_ substrates, obtained at room and cryogenic temperatures.

## Graphical abstract

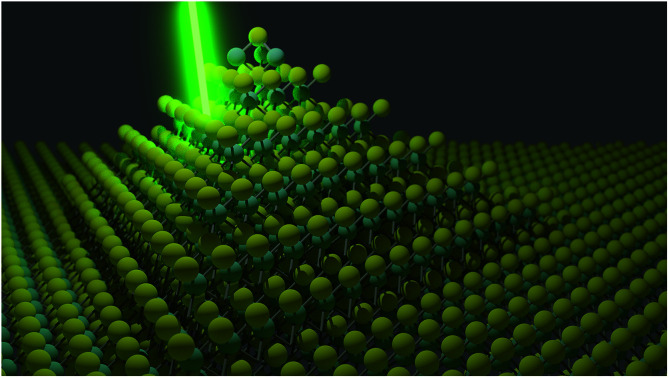

## Introduction

In the last decade, the rise of monolayer (ML) transition metal dichalcogenides (TMDs) has changed the paradigm for the coupling of two-dimensional materials to a well-established platform without the constraints imposed by epitaxies such as crystal-lattice match or chemistry compatibility. The employment of semiconducting monolayers has resulted in several applications in different fields such as electronics,^1,2^ valleytronics,^3^ energy storage,^4,5^ photovoltaics,^6^ light detection^7^ and chemical sensing.^8^

Among TMDs, molybdenum disulfide (MoS_2_) has been demonstrated to be the most versatile material for optoelectronic and photonic applications due to its layer-dependent optical properties.^9,10^ In the monolayer regime, MoS_2_ exhibits a direct optical bandgap of ∼1.8 eV in the visible range^11,12^ and an exciton binding energy of ∼1eV,^13^ which makes it a good candidate for novel applications ranging from photonics to optoelectronics.^7^ Although the main focus was devoted to monolayer MoS_2_, twisted bilayer MoS_2_ has also demonstrated interesting optical properties related to angle-dependent interlayer coupling.^14–16^

In the few-layer and multi-layer regimes, MoS_2_-based optoelectronic devices have gained an increasing interest thanks to enhanced light absorption, fast optical switching, saturable absorption, near-infrared emission and broadband spectral detection.^17–23^

Among the possible three-dimensional structures, MoS_2_ pyramids have demonstrated promising properties for light emission, non-linear optics, such as second-harmonic generation, and ferromagnetism.^24–26^

In this work, we show the excitonic absorption and cathodoluminescence (CL) emission in MoS_2_ micro-pyramids grown by chemical vapor deposition (CVD) on SiO_2_ substrates. The excitonic absorption was obtained at room and cryogenic temperatures by taking advantage of the cathodoluminescence emission of the SiO_2_ substrate. The CL emission is localized at the pyramid edges with an enhanced intensity at the pyramid basal vertices. By comparing the photoluminescence and absorption analyses, it was possible to obtain the MoS_2_ pyramid Stokes shift. This particular analysis is a completely novel method for analysis at cryogenic temperatures and the nanoscale spatial resolution of the technique. It is also possible to obtain the temperature-related shifts of the excitonic absorptions of the MoS_2_ pyramid.

**Fig. 1:**
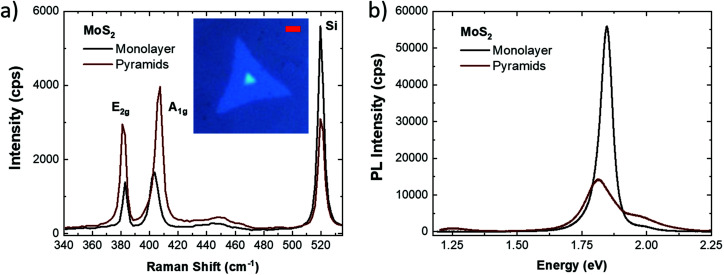
(a) Raman and (b) PL spectra of the MoS_2_ monolayer (black line) and 3D pyramids (red line), respectively. The inset presents the optical image of the MoS_2_ monolayer where a 3D pyramid is set at the center. The scale bar is 4 μm.

Fig. 1a presents the comparison of the Raman spectra of the MoS_2_ monolayer and three dimensional (3D) pyramids. The inset reports the optical micrograph of a triangular MoS_2_ monolayer where the 3D pyramid is set at the center of the flake with 0° twisting with respect to the surrounding monolayer; the optical contrast suggests the bulk nature of the pyramid. The morphological analysis of the pyramid by atomic force microscopy (AFM) is reported in Fig. S1.† The central position of the pyramid suggests that this structure is the seeding point of the monolayer MoS_2_. However, different studies on different TMDs show that the pyramid can originate in a layer-by-layer regime^27,28^ or a spiral growth regime due to the nucleation of screw dislocations during CVD growth.^26,29–31^ The Raman spectroscopic analysis confirms the bulk nature of the pyramid; in fact, the MoS_2_ E_2g_ and A_1g_ Raman modes appear at 381.7 cm^−1^ and 406.6 cm^−1^, with a separation of 24.9 cm^−1^, which is indicative of bulk MoS_2_.^32,33^ The surrounding triangular structure is in the monolayer form; in fact, the E_2g_ and A_1g_ modes, set at 382.8 cm^−1^ and 403.3 cm^−1^ respectively, have a 20.5 cm^−1^ separation, a signature of monolayer MoS_2_.^32,33^ In addition, the ratio between the MoS_2_ mode intensity and the silicon peak confirms the bulk nature of the pyramid. It is worth noting that the E_2g_/A_1g_ intensity ratio can give additional insights. The similar values, 0.84 and 0.74, in the case of the monolayer and pyramid, respectively, rule out the possible vertical growth of the MoS_2_ inside of the pyramid. Meanwhile, if we consider the A_1g_/E_2g_ intensity ratio as a benchmark of defect concentration (*i.e.* mainly sulfur vacancy),^23,32^ the pyramid presents a slightly higher value of 1.35 with respect to the 1.19 of the monolayer, which can indicate a higher concentration of defects in the three-dimensional structure. Fig. 1b presents the comparative analysis of the photoluminescence (PL) spectra of the MoS_2_ monolayer and the three-dimensional pyramid. The PL spectrum of the MoS_2_ ML shows a sharp peak at 1.85 eV with a shoulder on the high energy side; it is worth noting that the peak is slightly asymmetric, suggesting an additional emission on the low energy side. The pyramid spectrum presents a broader emission peak at 1.81 eV with a more prominent shoulder on the high energy side. Taking advantage of the Voigt deconvolution, reported in Fig. S2 of the ESI,† it is possible to highlight the presence of three different PL emissions. In particular, in the case of the MoS_2_ ML (Fig. S2a†), the three emissions are peaked at 2.00 eV, 1.846 eV and 1.79 eV, respectively; while in the PL spectrum of the MoS_2_ pyramid (Fig. S2b†), the three emissions are peaked at 1.92 eV, 1.81 eV and 1.73 eV, respectively. The two emissions at higher energies, at about 1.99 eV and 1.85 eV, are unequivocally attributed to the MoS_2_ B and A excitons,^11,12^ while the emission peak around 1.73 eV has possible different assignments, such as the trionic state^34,35^ or a defect-related intra-gap state.^36–38^

The redshifts of the excitonic light emissions are mainly related to the changes in thickness.^34^ It is worth noting that in the PL spectrum of the pyramid, a weak emission peak at 1.25 eV is found which is probably related to the indirect band-gap emission of MoS_2_.^23,39^ As expected, the A exciton emission is strongly suppressed in the MoS_2_ pyramid due to the bulk nature of the structure. However, a noticeable more intense emission of the B exciton is found in the 3D structure. The quenching of the A exciton is concurrent with the broadening of the A exciton emission; in fact, the full-width half maximum of the MoS_2_ ML increases from 0.03 eV up to 0.1 eV in the case of the pyramid.

**Fig. 2:**
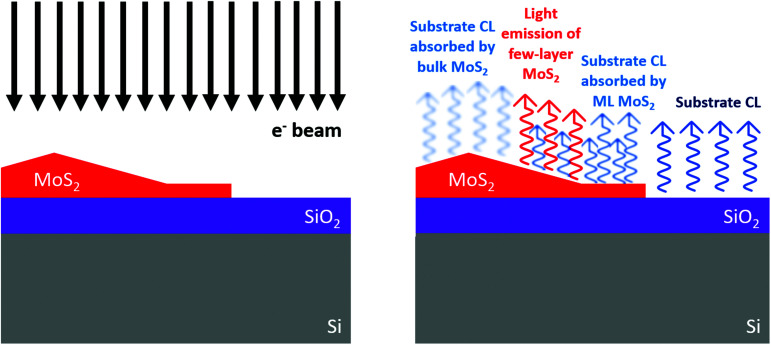
Schematic of the working principle of the proposed technique.

Fig. 2 shows the working principle of the technique employed for the analysis of the excitonic absorption and the possible CL emission. A bulk substrate topped with an MoS_2_ monolayer with a central pyramid is excited by an electron beam. The substrate emits light *via* the cathodoluminescence process. The emitted light is partially absorbed by the 2D material and is collected by a state-of-the-art CL system. A direct mapping of the substrate CL provides information on the light absorption by the 2D crystal with nanoscale resolution. CL is also able to probe the light emission of MoS_2_ few-layer and multi-layer regimes as previously reported.^23,40^ The result is a two-dimensional map of the absorption and emission that can reveal features hardly detectable using other electron microscopy or scanning confocal optical techniques. The main limitation to the spatial resolution is the interaction volume of the electron beam with the substrate. In fact, the spatial resolution is mainly dominated by the diameter of the interaction volume of the primary electron beam with the sample. The volume is dependent not only on the primary beam parameters, the accelerating voltage and the electron beam current, but also on the intrinsic properties of the analyzed sample, such as the sample density and the presence of the interfaces of different materials. The overall spatial resolution of this technique is estimated to be 200 nm (see Fig. S4† for the corresponding Montecarlo simulations). In addition, it is possible to obtain a CL absorption-emission spectrum, which is obtained by considering the cathodoluminescence emission of the substrate in the case of the analyzed object and the background. In this particular case, we obtained this spectrum by considering the CL spectra of the SiO_2_ topped with the MoS_2_ pyramid and the MoS_2_ ML as reference for the background surrounding the pyramid. In particular, the CL absorption-emission intensity as a function of the energy is calculated with the following formula:
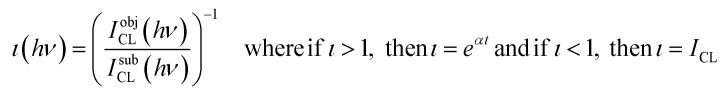
where *ι*(*hυ*) is the intensity of the absorption or the emission of the analyzed object that is obtained by the inverse ratio of the CL intensity of a desired object topped substrate (*I*^obj^_CL_) and the substrate's CL intensity (*I*^sub^_CL_). With this notation, if *ι*(*hυ*) is greater than 1, we have the absorption effect. As clarified in eqn (1), *ι*, in the case of the absorption effect, is equal to the exponent of the sample thickness (*t*) and the absorption coefficient (*α*). If the value of *ι*(*hυ*) is between 0 and 1, it represents the direct CL emission intensity of the object. This technique can be considered semi-quantitative, *i.e.* the *ι* value is proportional to a larger absorption of photons or a larger CL intensity. However, the limitation of this technique is the direct interaction of the primary electron beam with the 3D object on the surface. In fact, part of the electron scattering events, generating the CL signal of the substrate, occurs in the 3D object on the surface, causing a less-intense CL signal from the substrate. This is clarified by the Montecarlo simulations shown in Fig. S3 and S4.†

**Fig. 3:**
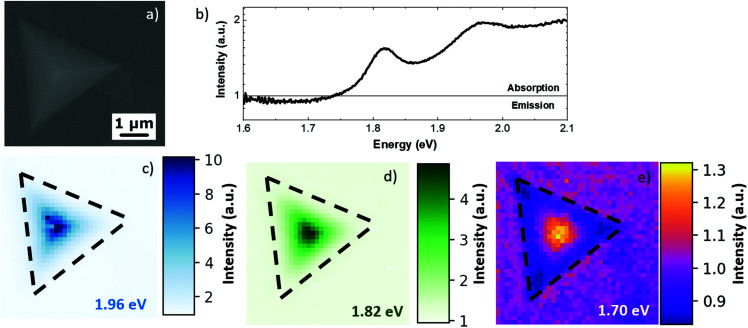
(a) SE micrograph of the MoS_2_ pyramid. (b) Absorption-emission spectrum obtained by the renormalization of the CL spectra obtained at room temperature, integrated over the whole pyramid. (c) Absorption map of exciton B (1.96 eV). (d) Absorption map of exciton A (1.82 eV). (e) CL absorption-emission map of the defect-related state (1.70 eV).

The SEM micrograph shown in Fig. 3(a) presents the MoS_2_ pyramid with a lateral size of 4 μm. The SEM image reveals that in this case the pyramid was grown in a layer-by-layer regime, as also supported by AFM analysis of Fig. S1.†^27,28^Fig. 3(b) presents the absorption-emission spectrum integrated over the whole pyramid. The spectrum presents two positive peaks and a negative band at 1.82 eV, 1.91 eV and 1.70 eV, respectively. The CL spectra of the pyramid and the substrate, from which the CL absorption-emission spectrum is obtained, are reported in Fig. S5 of the ESI.† The positive peaks are attributed to the absorption of the MoS_2_ A and B excitons, respectively, while the negative band is attributed to the light emission of the defect-related state excited by the electron beam.^36^ The 1.70 eV CL emission band is broad (about 0.15 eV), implying a possible convolution of the two different emissions related to defect states, ruling out the possible attribution of such emissions to trionic states. Compared with the PL analysis reported in Fig. 1, it is possible to evaluate the Stokes shift of the A and B excitons, which are 0.08 eV and 0.1 eV, respectively. The Stokes shift was previously reported in the case of the MoS_2_ ML on silicon dioxide substrates as 25 meV, which is mainly caused by the inhomogeneity of substrate-induced doping.^41^ We can exclude tensile strain as the cause of the large Stokes shift because increasing tensile strains normally induce a decrease of the Stokes shift as previously reported.^42,43^ Most likely, the cause of the large Stokes shift is the presence of a large density of defects in the MoS_2_ pyramid, which is in agreement with the CL emission.^44^ After acquiring the spectral information of the absorption peaks and emission bands, it was possible to map the intensity of the absorption and emission. Fig. 3(c)–(e) report the CL absorption-emission maps of excitons A and B and of the defect-related emission, respectively. The exciton A map shows an increasing absorption from the edge of the pyramid to the center of the pyramid; similarly, the map of exciton B shows analogous behavior. This effect reveals that the CL emission of the substrate is absorbed mainly upon increasing the thickness of the MoS_2_ pyramid, while the defect-related state map presents a light absorption at the center of the pyramid and direct CL emission at the edges. The localized light emission at the pyramid edges can be attributed to different effects: a larger concentration of defects at the pyramid edges;^23^ the formation of a whispering-gallery-mode cavity related to the partial destructive interference of nonlinear polarizations between the neighboring atomic layers, as previously reported in the case of the WS_2_ pyramid, grown in the layer-by-layer regime;^28^ or the modification of the MoS_2_ band structure at the Γ point at the few-layers limit^12^ (the pyramid edges are 2 nm thick, as evidenced by AFM analysis) that creates a direct radiative recombination path with the defect-related intra-gap state.

Normally, the absorption properties of 2D materials are analyzed by means of transmittance and reflectance spectroscopy techniques,^45,46^ which are the benchmarks used for such properties. However, the spatial resolutions of these techniques are limited to the size of the optical probe. Thanks to the QNAM (Quantitative Nanoscale Absorption Mapping) technique, we can overcome that drawback by taking advantage of the use of electron beam probes.^47^ Recently, spatially resolved absorption mapping has mainly demonstrated the evolution of the absorption properties of TMDs by increasing the number of layers in exfoliated flakes.^48,49^ In addition, most of the reports of transmittance and reflectance spectroscopic techniques are limited to room temperature operations. An additional mapping technique that can provide quantitative information of the optical properties is imaging spectroscopic ellipsometry (ISE). In general, ISE can provide access to the local dielectric function of two-dimensional materials on a large spectral range (from deep UV down to NIR).^50,51^ With respect to QNAM, ISE has shortcomings: (1) ISE is an indirect measurement, *i.e.* in general, the measured Ψ and Δ values cannot be converted directly to the optical constants of the sample.^52^ Normally, a model analysis must be performed with detailed knowledge of the sample structures. (2) ISE spatial resolution is still limited to the micrometric range.^51^ QNAM's main limitation, instead, is that the substrate should have CL emissions in the optical spectral range of interest.

**Fig. 4:**
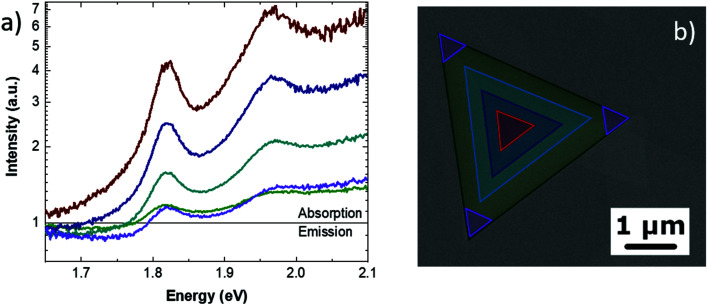
(a) Absorption–emission spectra obtained at different regions of the pyramid (b) The SEM micrograph of the highlighted regions, with the same color code as the spectra, where the spectra are obtained by signal integration.

By integrating the optical signals from particular regions of the pyramid, it is possible to obtain more detailed information. Fig. 4(a) presents the optical spectra obtained from the regions of the pyramid highlighted by triangles in Fig. 4(b) with the same color code of the spectra. The direct comparison of the different areas reveals that the center of the pyramid solely presents the highest absorption of both the A and B excitons, with no defect emission. Furthermore, it is interesting to observe that the comparison of the spectra, integrated into different areas of the pyramid, reveals that the absorption peaks do not show any shift related to the MoS_2_ thickness. Moving from the center to the edges of the pyramids, the absorption intensity decreases. In particular, the spectrum obtained at the edge of the pyramid presents a faint absorption with a broadening of both of the excitonic peaks. Particularly noteworthy is that the highest intensity of the defects emission is detected at the base vertex of the pyramid (purple line in Fig. 4(a). This enhancement can be attributed to the formation of a whispering-gallery-mode cavity. The CL spectra of the different regions and the integration masks are reported in Fig. S5 of the ESI.†

**Fig. 5:**
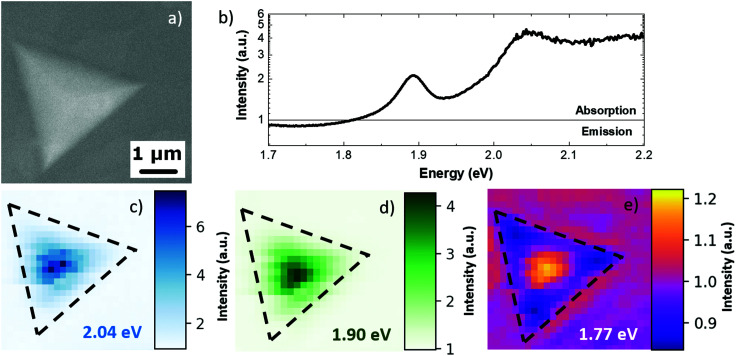
(a) SE micrograph of the MoS_2_ pyramid at *T* = 10K. (b) CL absorption-emission spectrum at the cryogenic temperature. (c) Absorption map of exciton B (2.04 eV). (d) Absorption map of exciton A (1.90 eV). (e) CL absorption-emission map of the defect-related intra-gap state (1.77 eV).

Fig. 5 presents the CL absorption-emission analysis carried out at the cryogenic temperature (*T* = 10 K) on the same pyramid shown in Fig. 3. Fig. 5a shows the SEM image of the pyramid in analysis. The cryogenic temperature CL absorption-emission spectrum, integrated over the whole pyramid, is shown in Fig. 5b. Similar to the room temperature analysis, the spectrum presents two peaks related to the absorption of the A and B excitons of MoS_2_ and a valley related to the defect-related light emission. The A and B exciton absorption peaks appear at 1.89 eV and 2.04 eV respectively. The light emission assigned to defects is a dip at 1.77 eV. By comparing the cryogenic temperature analysis with the room temperature analysis, it is possible to evaluate the temperature-related shift of the excitonic absorption peaks. The temperature-related blue shift is 0.07 eV and 0.13 eV for the A and B excitons, respectively. These values are in good agreement with previous temperature-related shifts of absorption peaks for different TMDs.^53,54^ It is worth noting that the light emission related to the defects presents a temperature shift of 0.05 eV. The CL spectra are reported in Fig. S6.†Fig. 5c and d present the absorption maps of the A and B excitons, respectively. The absorption increases from the pyramid edge and toward the pyramid center. The defect-related emission, Fig. 5e, shows that the center of the pyramid presents a light absorption while the edge of the pyramid presents a faint emission. The emission is contrarily enhanced at the base vertices of the pyramid, similar to the room temperature analysis.

**Fig. 6:**
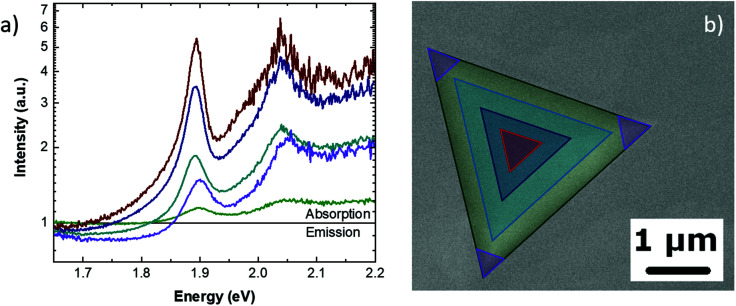
(a) Cryogenic temperature absorption-emission spectra obtained at different regions of the pyramid. (b) The SEM micrograph of the highlighted regions, with the same color code as the spectra, where the spectra are obtained by signal integration.

With the same integration procedure as shown in Fig. 4, it is possible to obtain the CL absorption-emission spectra of different areas of the MoS_2_ pyramid (Fig. 6). This analysis reveals that the absorption effect is stronger at the center of the pyramid and becomes fainter at the pyramid edges. It is worth noting that by comparing the room and cryogenic temperature analyses, the absorption at the pyramid edges is suppressed at the cryogenic temperature while the defect-state emission results are enhanced at the pyramid vertices. The defect-related emission is localized close to the pyramid vertices. The CL spectra and the integration masks are reported in Fig. S7.†

## Conclusion

In conclusion, we show the excitonic absorption and cathodoluminescence (CL) emission of MoS_2_ micro-pyramids grown by chemical vapor deposition (CVD) on SiO_2_ substrates. The excitonic absorption was obtained at room and cryogenic temperatures by taking advantage of the cathodoluminescence emission of the SiO_2_ substrate. The defect-related CL emission is localized at the pyramid edges with an enhanced intensity at the pyramid basal vertices. By comparing photoluminescence and absorption analyses, it is possible to obtain the MoS_2_ pyramid Stokes shift of both the A and B excitons. This particular analysis is a completely novel method for the analysis at cryogenic temperatures and the nanoscale spatial resolution of the technique. It is also possible to obtain the temperature-related shifts of the A and B exciton absorption of the MoS_2_ micro-pyramid.

## Methods

### MoS_2_ Monolayers on SiO_2_/Si substrates

Monolayers of MoS_2_ were grown by CVD on a *p*-type Si substrate with a 300-nm thick SiO_2_ layer at a temperature of ∼715 °C. The substrate was placed face-up in the center of a tube furnace on a combustion boat with 10 mg of MoO_3_ powder spread along the bottom. An identical boat containing 1 g of sulfur was placed at a position upstream at the opening of the furnace such that the maximum temperature at that position was 250 °C. After the tube was sealed, ultra-high purity argon gas was allowed to flow at room temperature for 10 min in order to purge any remaining oxygen. The furnace was then heated to 715 °C over a period of 25 min at which point the temperature was held constant for an additional 15 min. The furnace was then allowed to cool back to room temperature naturally over a period of ∼90 min. During the entire growth period, the flow rate of argon was 50 sccm. For the post-growth annealing experiments, one sample was subdivided into three and they were then placed on quartz slides in a tube furnace and annealed at 150 °C, 200 °C, and 250 °C, separately, for 1 h in Ar flowing at 50 sccm. The samples were cooled naturally to room temperature following the annealing process.

### Raman and PL spectroscopy

Raman and photoluminescence (PL) spectroscopy were carried out with a Renishaw inVia system, equipped with a confocal microscope, a 532 nm excitation laser and an 1800 line/mm grating (spectral resolution 2 cm^−1^). All of the analyses were performed with the following parameters: an excitation laser power of 500 μW, an acquisition time of 4 s for each spectrum, and a spot size of 800 nm with a 100X objective (NA = 0.85). The uncertainty of the PL peak position is 0.05 nm.

### CL hyperspectral mapping

An Attolight Rosa SEM-CL microscope was used for the absorption/emission experiments. The experiments are standard CL maps, where the CL signal is employed to evaluate the absorption of the two-dimensional material on top of the light-emitting substrate. For the semi-quantitative comparison between different areas of the sample, such as between bare and coated substrate regions, the intensity is normalized over the number of pixels that compose each area. All of the data were obtained at room and cryogenic temperatures (*T* = 10 K) under UHV conditions. The e-beam current is about 1 nA with an accelerating voltage of 5 kV, resulting in an electron beam power of *P* = 5 × 10^−6^ W. The acquisition time for each spectrum was1s at room temperature and 0.7 s at the cryogenic temperature. Considering that the RT map is 40 × 40 pixels, the map acquisition time was 27 minutes, while the 10 K map was 36 × 36 pixels resulting in an acquisition time of 15 minutes. The Attolight Rosa SEM-CL is a dedicated instrument that implements cathodoluminescence (CL) detection as a reflective microscopy objective embedded within the pole piece. This allows for CL acquisitions to encompass large areas without a loss of signal and with a high collection efficiency. In particular, electrons from the electron gun are collimated by the gun lens and then focused by the SEM objective lens on the sample. The CL objective and tilted mirror ensemble, embedded into the objective lens, gather the CL signal and focus it into the spectrometer. The CL signal is sent to a spectrometer with a focal length of 32 cm by means of an objective (N.A. 0.71) placed in the electron microscope. The system is equipped with a Peltier-cooled charge-coupled device (CCD) and a 600 line per mm diffraction grating.

## Conflicts of interest

There are no conflicts to declare.

## Supplementary Material

NR-014-D1NR06041D-s001
