# Supplementary material for: Excitonic absorption and defect-related emission in three-dimensional MoS2 pyramids
Source: Nanoscale. 2021 Dec 17;14(4):1179–86. doi: 10.1039/d1nr06041d (PMC8793919; doi:10.1039/d1nr06041d)
Supplement: NR-014-D1NR06041D-s001 [file NR-014-D1NR06041D-s001.pdf]

Electronic Supplementary Material (ESI) for Nanoscale.
This journal is © The Royal Society of Chemistry 2021

Supporting Information

Excitonic absorption and defect related emission in three-dimensional MoS_2 pyramids

M. Negri*[1,2], L. Francaviglia[1+], D. Kaplan[3], V. Swaminathan[3,4], G. Salviati[2], A. Fontcuberta i Morral[1,5], F. Fabbri*[6]

[1] Institute of Materials, Faculty of Engineering, École Polytechnique Fédérale de Lausanne, 1015 Lausanne, Switzerland
[2] Institute for Materials for Electronics and Magnetism (IMEM-CNR), Parco Area delle Scienze 37/A, 43124 Parma, Italy
[3]U.S. Army RDECOM-ARDEC, Fuze Precision Armaments and Technology Directorate, Picatinny Arsenal, NJ 07806, USA
[4] Department of Physics, Penn State University, USA.
[5] Institute of Physics, Faculty of Basic Sciences, École Polytechnique Fédérale de Lausanne, 1015 Lausanne, Switzerland
[6] NEST, Istituto Nanoscienze - CNR, Scuola Normale Superiore, Piazza San Silvestro 12, 56127 Pisa, Italy
+ Current address: Molecular Foundry, Lawrence Berkeley National Laboratory, Berkeley, CA 94720, USA
* Current address: Axcelis technologies, Agrate Brianza (MB), Italy

*Corresponding authors: M. Negri negri.m1@gmail.com

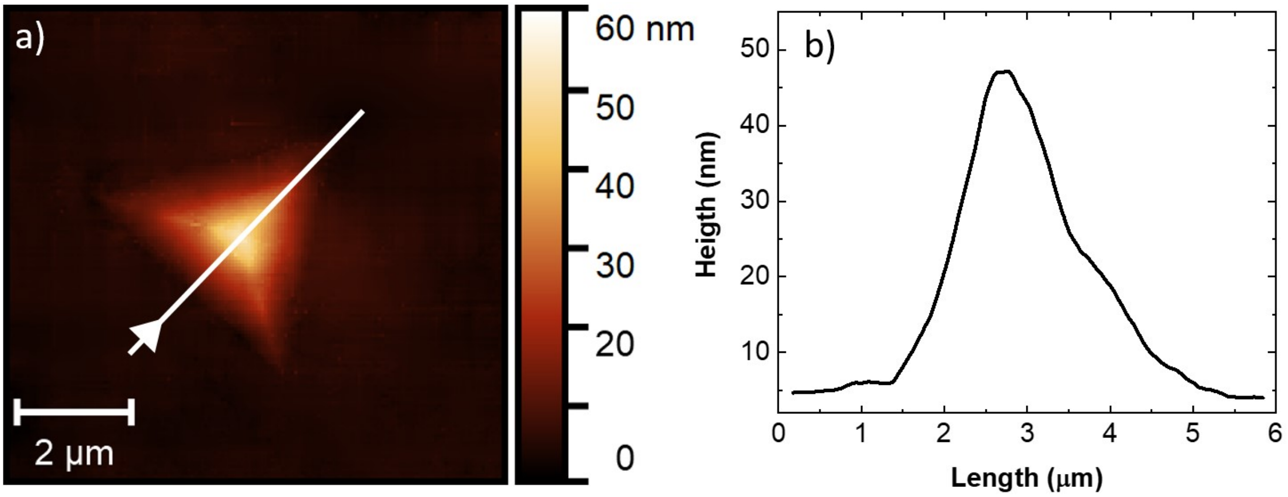

S1 Morphological analysis of the MoS_2 pyramid carried out by atomic force microscopy. In particular, a) the thickness map of the pyramid, the white line indicated where the height profile is extracted from the map and reported in panel b).

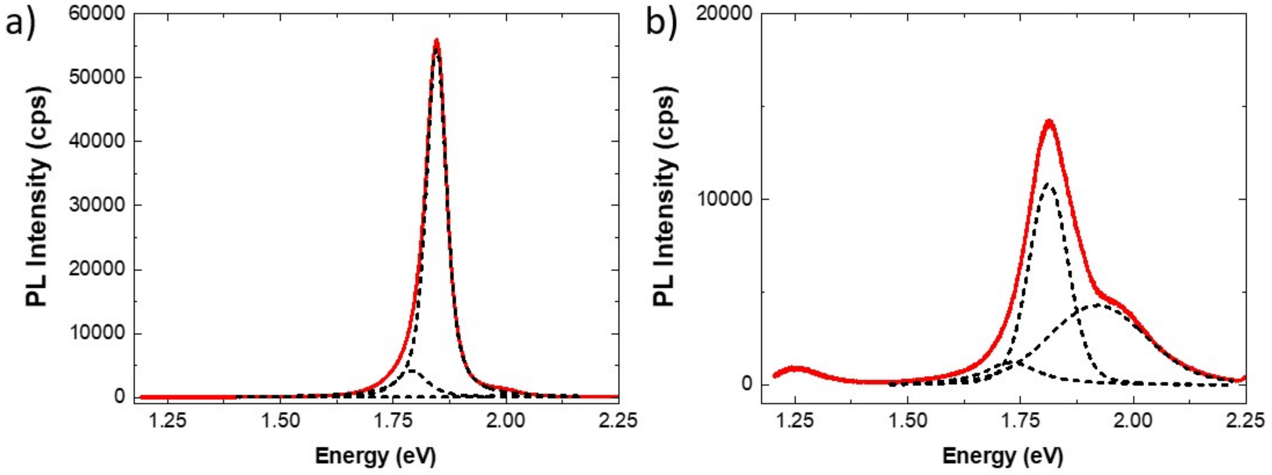

Figure S2 Voigt peak deconvolution of PL spectra

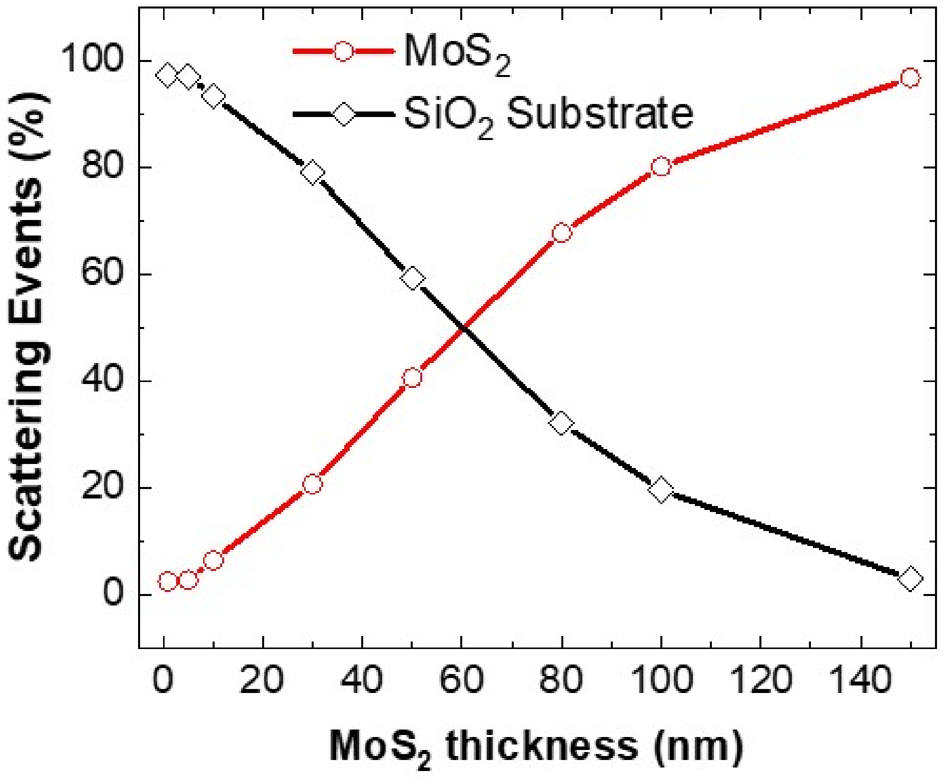

Figure S3 Scattering Events in MoS_2 and in the SiO_2 substrate with increasing thickness of the MoS_2 layer.

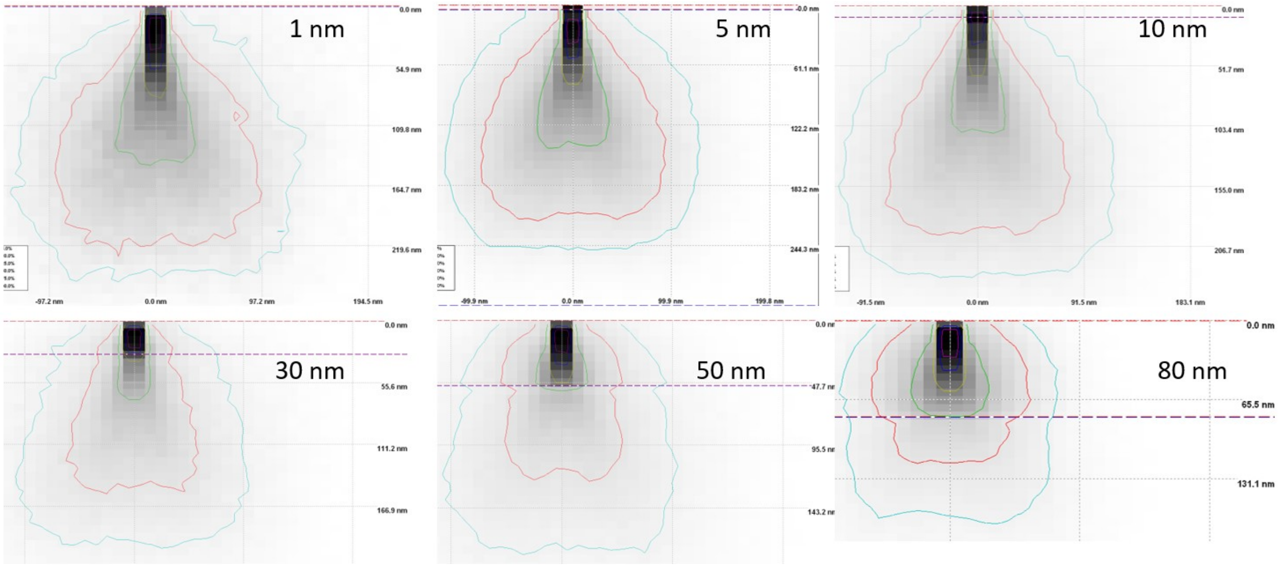

Figure S4 Energy release diagram of the electron beam for increasing MoS_2 thickness. Considering the generation/recombination volume the electron beam power density can be evaluated in $1.2x10^9$ W/cm^3.

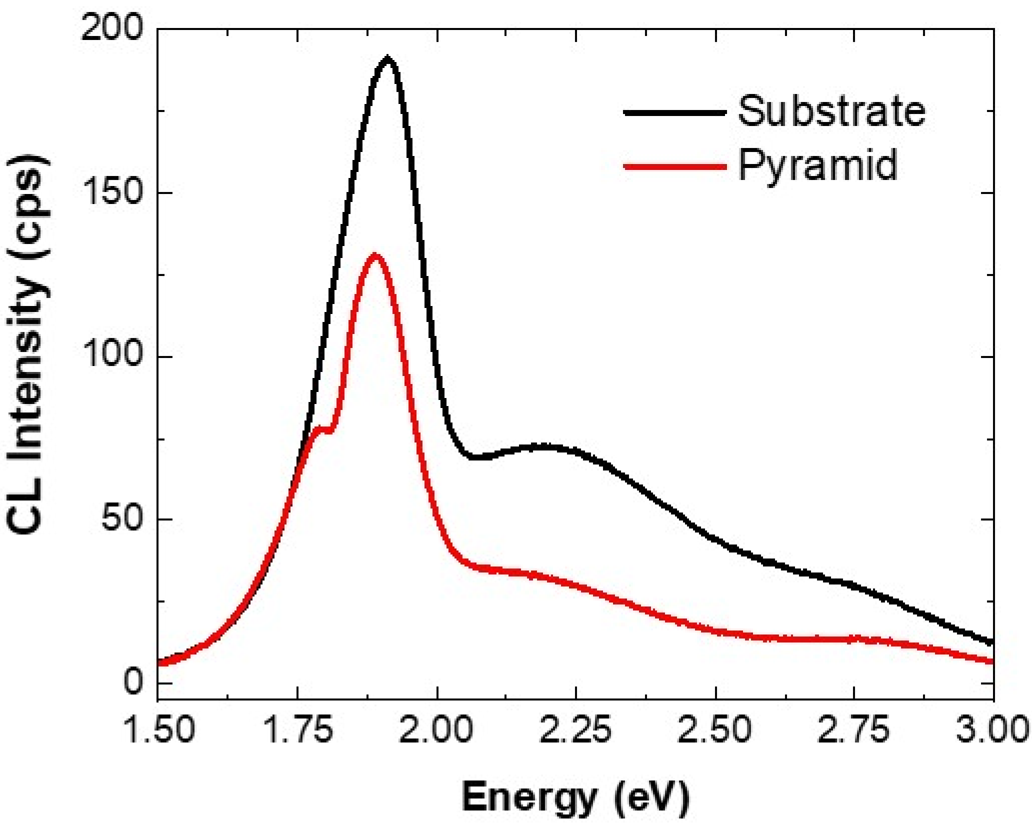

Figure S5 Room temperature CL spectra, from which the absorption / emission spectrum of Figure 3 is obtained.

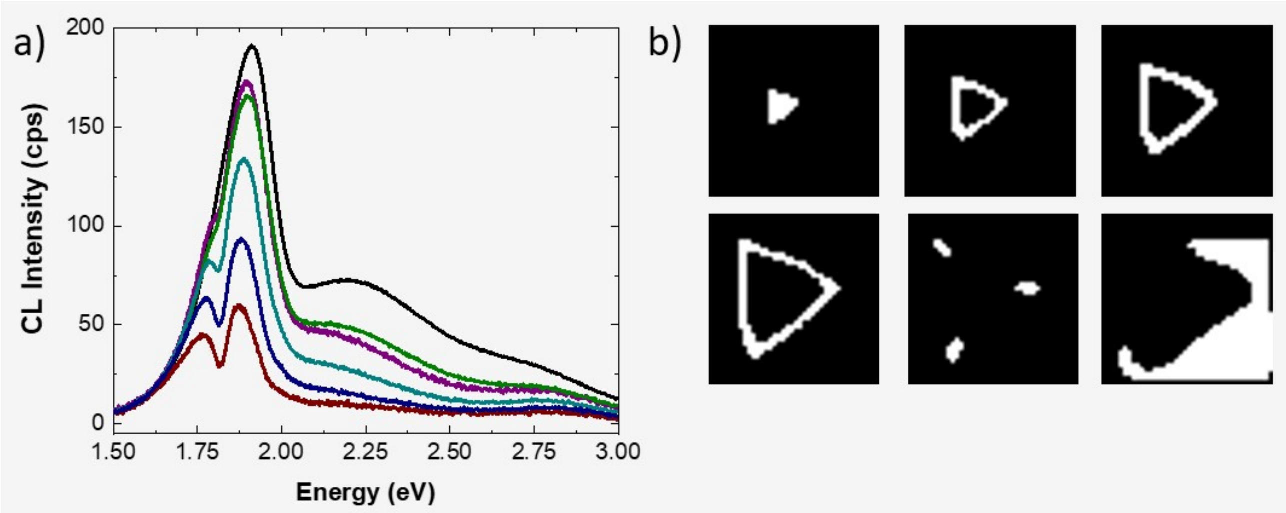

Figure S6 Room temperature CL spectra, from which the absorption / emission spectrum of Figure 4 are obtained. b) Integration masks for the areas where the spectra are obtained.

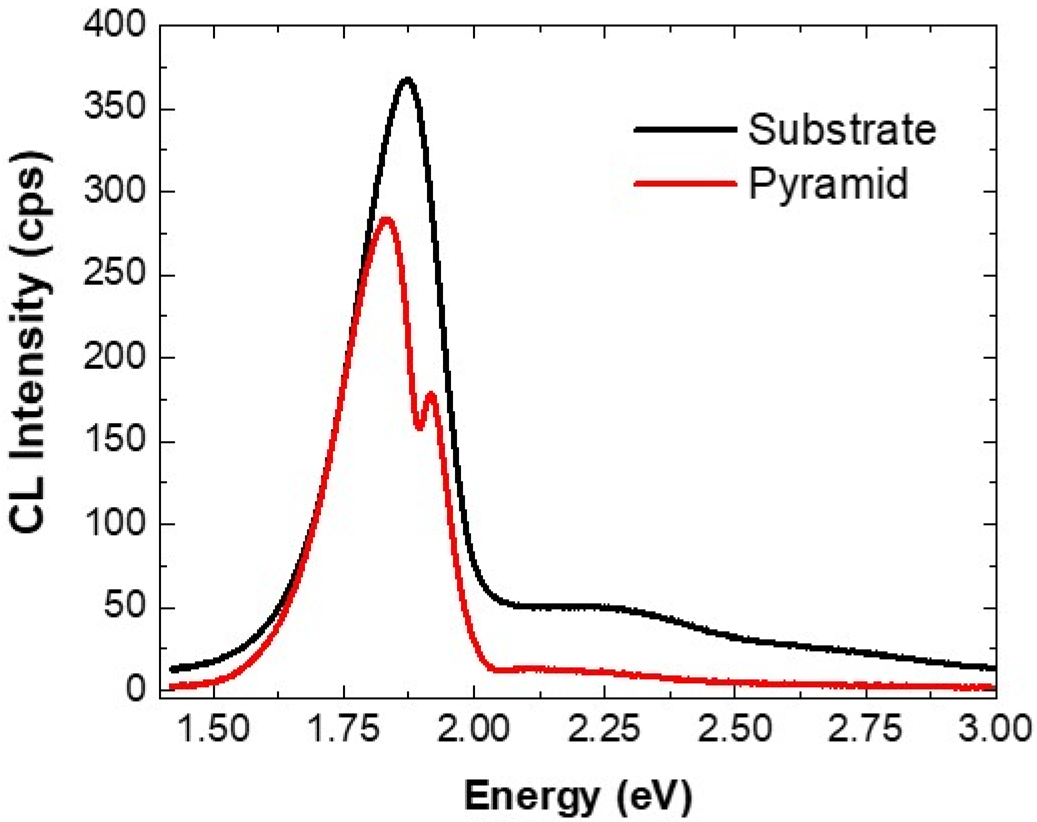

Figure S7 Cryogenic temperature CL spectra, from which the absorption / emission spectrum of Figure 5 is obtained.

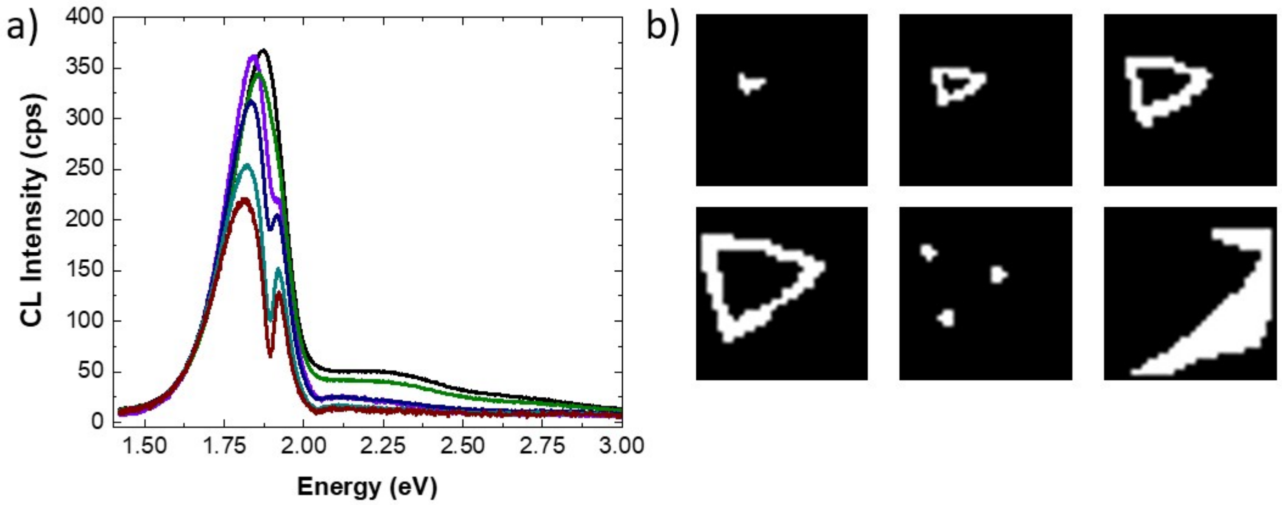

Figure S8 Cryogenic temperature CL spectra, from which the absorption / emission spectrum of Figure 4 are obtained. b) Integration masks for the areas where the spectra are obtained.
